# Intrathecal [^64^Cu]Cu-albumin PET reveals age-related decline of lymphatic drainage of cerebrospinal fluid

**DOI:** 10.1038/s41598-023-39903-y

**Published:** 2023-08-09

**Authors:** Azmal Sarker, Minseok Suh, Yoori Choi, Ji Yong Park, Yun-Sang Lee, Dong Soo Lee

**Affiliations:** 1 Department of Nuclear Medicine, College of Medicine, Seoul National University, Seoul, Korea; 2 Department of Nuclear Medicine, Seoul National University Hospital, Seoul, Korea; 3 Biomedical Research Center, Seoul National University Hospital, Seoul, Korea; 4 Department of Molecular Medicine and Biopharmaceutical Sciences, Graduate School of Convergence Science and Technology, Seoul National University, Seoul, Korea; 5 Medical Research Center, College of Medicine, Seoul National University, Seoul, Korea; 6 Medical Science and Engineering, School of Convergence Science and Technology, Pohang University of Science and Technology (POSTECH), Pohang, Korea

**Keywords:** Neuroimmunology, Ageing, Neurophysiology

## Abstract

Age-related cognitive decline is associated with dysfunctional lymphatic drainage of cerebrospinal fluid (CSF) through meningeal lymphatic vessels. In this study, intrathecal [^64^Cu]Cu-albumin positron emission tomography (PET) was applied in mice to evaluate lymphatic drainage of CSF and its variation with age. [^64^Cu]Cu-albumin PET was performed at multiple time points after intrathecal injection of [^64^Cu]Cu-albumin at an infusion rate of 700 nl/min in adult and aged mice (15–25 months old). CSF clearance and paravertebral lymph nodes were quantified after injection and during the stationary phase. Stationary phase of the next day followed the initial perturbed state by injection of 6 ul (1/7 of total CSF volume) and CSF clearance half-time from the subarachnoid space was 93.4 ± 19.7 and 123.3 ± 15.6 min in adult and aged mice (p = 0.01), respectively. While the % injected dose of CSF space were higher, the activity of the paravertebral lymph nodes were lower in the aged mice on the next day. [^64^Cu]Cu-albumin PET enabled us to quantify CSF-lymphatic drainage across all levels of brain spinal cords and to visualize and quantify lymph node activity due to CSF drainage. [^64^Cu]Cu-albumin PET revealed the age-related decrease of the lymphatic drainage of CSF due to this decreased drainage from the subarachnoid space, especially during the stationary phase, in aged mice.

## Introduction

Since the discovery of the meningeal lymphatics in 2015^1,2^, the interstitial fluid (ISF)—cerebrospinal fluid (CSF)—meningeal lymphatic drainage pathway has been considered the major route for brain and spinal cord waste disposal. This pathway is also the route whereby leukocytes enter the CSF and consequently brain parenchyma and spinal cord via pia or ependyma lining the brain and spinal cord^3,4^. Mice and rats use their dual pathway of drainage via the nasal cavity and meningeal lymphatics for waste disposal^5–7^, whereas humans mainly use the dura lymphatics, not the nasal cavity^8–11^. ISF passes through glia limitans to the perivascular space, which communicates with the CSF space. Thus, any waste in ISF is drained to the dura meningeal lymphatics of the brain and spinal cord^12^. This function and dysfunction of glymphatic drainage^13^, has been investigated in rodents using fluorescent macromolecules^14^ and in humans using gadolinium contrast magnetic resonance imaging (MRI)^8^.

Glymphatic/lymphatic imaging has been performed using contrast administration via cisterna magna and fluorescent microscopy or MRI in rodents^12,15–17^ or using L4/5 intervertebral intrathecal administration and MRI in humans^8–11^. Both approaches enable us to visualize the reflux of CSF to the perivascular space to evaluate glymphatic function and lymphatic drainage of CSF. In our preliminary study, we found that intra-cisterna magna administration of [^64^Cu]Cu-albumin was too invasive to unveil the subtle changes in the kinetics of lymphatic drainage of CSF, requiring optimizing the quantity and volume of tracer^18^. Unlike the initial reports that revealed glymphatic function using fluorescence microscopy in rodents^12,15,17,19^, recent reports have primarily used intrathecal MRI contrast agents, showing minute differences in the reflux from the submeningeal to the perivascular space^20–22^ and thus preventing the use of intrathecal contrast MRI to evaluate glymphatic function. In contrast, the lymphatic drainage of CSF could be studied using intrathecal contrast MRI and intrathecal cisternography positron emission tomography (PET)^18^, with high contrast and without background, thus demonstrating the superior performance in imaging the primary/secondary sentinel lymph nodes down the skull and along the vertebral columns in rodents^21–23^.

The contribution of the lymphatic drainage of CSF is now known to be mandatory for central nervous system (CNS) health because waste disposal is crucial for maintaining the brain/spinal cord function. This lymphatic drainage is particularly important during slow wave sleep and anesthesia (reviewed in^24^), which has incremental value further to the in situ clearance of the debris/waste by the glial cells. Dysfunctions beyond the physiologic range of variation associated with sleep, tend to be subtle and minute as observed in age-related changes in the lymphatic drainage of CSF^25–31^. Thus, the volume of intrathecal contrast must have been minimized to avoid disturbing the physiological stationarity, but large enough for CSF circulation to deliver the administered tracers along the CSF flow and finally to the outside of the CNS. CSF starts flowing from the ventricular choroid plexus, continuing through the aqueduct/spinal canal and cisterns, rounding and soaking perivascular spaces via the entire subarachnoid space. Then, CSF passes through the arachnoid barrier cell layer of the brain and spinal cord to reach the meningeal extracellular space, eventually entering the lymphatic channels of the cranial/vertebral dura via various pathways such as perineural and dural gaps^32^. Several radionuclide/radiopharmaceuticals, which have been clinically used for radioisotope cisternography for decades, could be used for imaging the lymphatic drainage of CSF. In our preliminary study, we chose [^64^Cu]Cu-albumin and established [^64^Cu]Cu-albumin PET protocols for imaging lymphatic drainage of CSF in mice^18^. This approach also visualized primary/secondary sentinel lymph nodes down the skull along the vertebral column in mice by [^64^Cu]Cu-albumin PET^18^.

In this study, we corroborated the findings of our previous study, using the aforementioned protocol in a simple mouse aging model and assessed the variations in the lymphatic drainage of CSF with aging using intrathecal [^64^Cu]Cu-albumin PET to visually and quantitatively analyze CSF clearance, its temporal progress after injection, and lymph node retention immediately after injection and during the stationary phase. Mouse CSF, which replenishes 10 or more times per day, was perturbed by injecting 6 μl (1/7th of the total CSF volume) of [^64^Cu]Cu-albumin within 9 min. After resting for a day, the mice were imaged again for assessing the stationary CSF-lymphatic drainage and lymph node activity under continuous influx/drainage of its own.

## Materials and methods

### Animals

Male C57BL/6 mice at 2–9 months of age were used as the adult, whereas those at 15 to 25 months of age were used as the aged mice^33^; 2–5 mice were housed per cage with access to standard food and potable water ad libitum. The housing room was maintained at a constant temperature of 22–24 °C with a 12/12-h light–dark cycle. A Y-maze test was conducted to assess behavioral differences between groups. This study shares an animal cohort with previously published literature^18^, which established the [^64^Cu]Cu-albumin PET protocol for evaluating lymphatic drainage of CSF in adult mice. Animal experiment was approved by Institutional Animal Care and Use Committee (IACUC) of Seoul National University (SNU-200513-8-6) and all methods were carried out in accordance with relevant IACUC guidelines and regulations of Seoul National University and ARRIVE guidelines (https://arriveguidelines.org). Relevant details are in Supplemental Notes.

### Y-maze test

Behavioural test was performed in a Y-shaped maze under dim lighting. For adaptation of this test, mice were introduced into the center of the maze and allowed to visit all three arms the day before testing. For Y-maze test, a mouse was introduced into the center of the maze and allowed to freely explore the maze for 8 min. To calculate the alternation, visits of each arm were recorded when the limb and tail were within an arm. The alternation percentage was calculated by dividing the number of sequential alternations of the three arms by the total number of alternations possible.

### [^64^Cu]Cu-albumin radiolabeling

Human serum albumin, with a molecular weight of 66.5 kDa, was radiolabeled with [^64^Cu]Cu (t1/2 = 12.7 h, β^+^  = 655 keV 17.8%, β^‒^ = 579 keV 38.4%) using a click chemistry-based method^34^. Briefly, (1) ^64^Cu in HCl solution was dried with a nitrogen flow of 5–10%, (2) 1 M sodium acetate buffer was added to adjust the pH to 5.3, (3) *p*-isothiocyanatobenzyl-1,4,7-triazacyclononane-1,4,7-triacetic acid (NOTA)-N_3_-1300 nmol/mL, 10 μL was added, and the mixture was placed in a heat block at 60–70 °C for 10 min, (4) labeled NOTA-N_3_ of [^64^Cu]Cu NOTA-N_3_ was added to 1 mg/mL of azadibenzocyclooctyne (ADIBO)-albumin (human serum albumin, 66.7 kDa) and placed in an orbital shaker for 10 min, (5) the mixture was filtered and then centrifuged at 15,000 rpm for 5 min. The labeling efficiency of [^64^Cu]Cu-albumin was 99%, as confirmed by instant thin layer chromatography (ITLC), with 0.1 M citric acid solution as a developing solvent.

### Intrathecal [^64^Cu]Cu-albumin injection and PET image acquisition

Intrathecal administration followed the procedure used for preclinical or clinical radioisotope cisternography^18^. Briefly, an intrathecal injection was performed after palpation of the L4 spine. A 1-cm-long sagittal incision was made, and a needle was inserted through the muscles up to an appropriate length while waiting for the tail to flick. [^64^Cu]Cu-albumin injection was performed using a syringe pump (Harvard Apparatus) at an infusion rate of 700 nl/min, injecting a total volume of 6 μL with an amount of radioactivity ranging from 0.74 to 1.85 MBq. The needle was kept in place until the first imaging session was over. Then, the needle was removed, and the wound was closed.

Genisys PET box (Sofie Biosciences) was used for whole-body PET acquisition of static PET in list mode for 6 min at each of the image acquisition time points, which were 9 min, 2, 4, 6, 12, and 24 h post-injection for adult mice, and 9 min, 2, 4, 6, 17, and 24 h post-injection for aged mice. Injection and image acquisition were performed under isoflurane anesthesia, with 3% for induction, and 2.8% for maintenance with 500 ml/min oxygen inhalation.

### Visual image analysis and quantitation

Using the MIM software, 3D regions of interest (ROI) were drawn, and counts were measured for the entire subarachnoid space. Subarachnoid space (Supplementary Fig. 1) counts of 9 min (h_0_) were used as a surrogate of the injected dose for normalization of the counts of all other ROIs at any given time point (h_n_), which were expressed as %ID. The %ID of each time point (h_n_/h_0_) was plotted against the corresponding time points to generate the time-%ID curves. Using the one-compartment exponential model of GraphPad Prism, the half-life (t_1/2_) was calculated for each mouse. The coefficient of determination R^2^ > 0.95 showed a good fit. Unpaired t-test with Welch’s correction was used to compare half-lives between groups.

### Assessment of in-vivo stability of [^64^Cu]Cu-albumin

CSF was collected from an adult mouse, one and four hours after intrathecal injection of [^64^Cu]Cu-albumin, and ITLC was performed using the routine protocol.

## Results

### Behavioral differences between adult and aged mice

The Y-maze test revealed a significant decline (Mann Whitney, p < 0.05) in the spatial memory among aged mice in comparison with adult mice (Supplementary Fig. 2).

### Visual comparison of intrathecal [^64^Cu]Cu-albumin PET images in adult and aged mice

**Figure 1:**
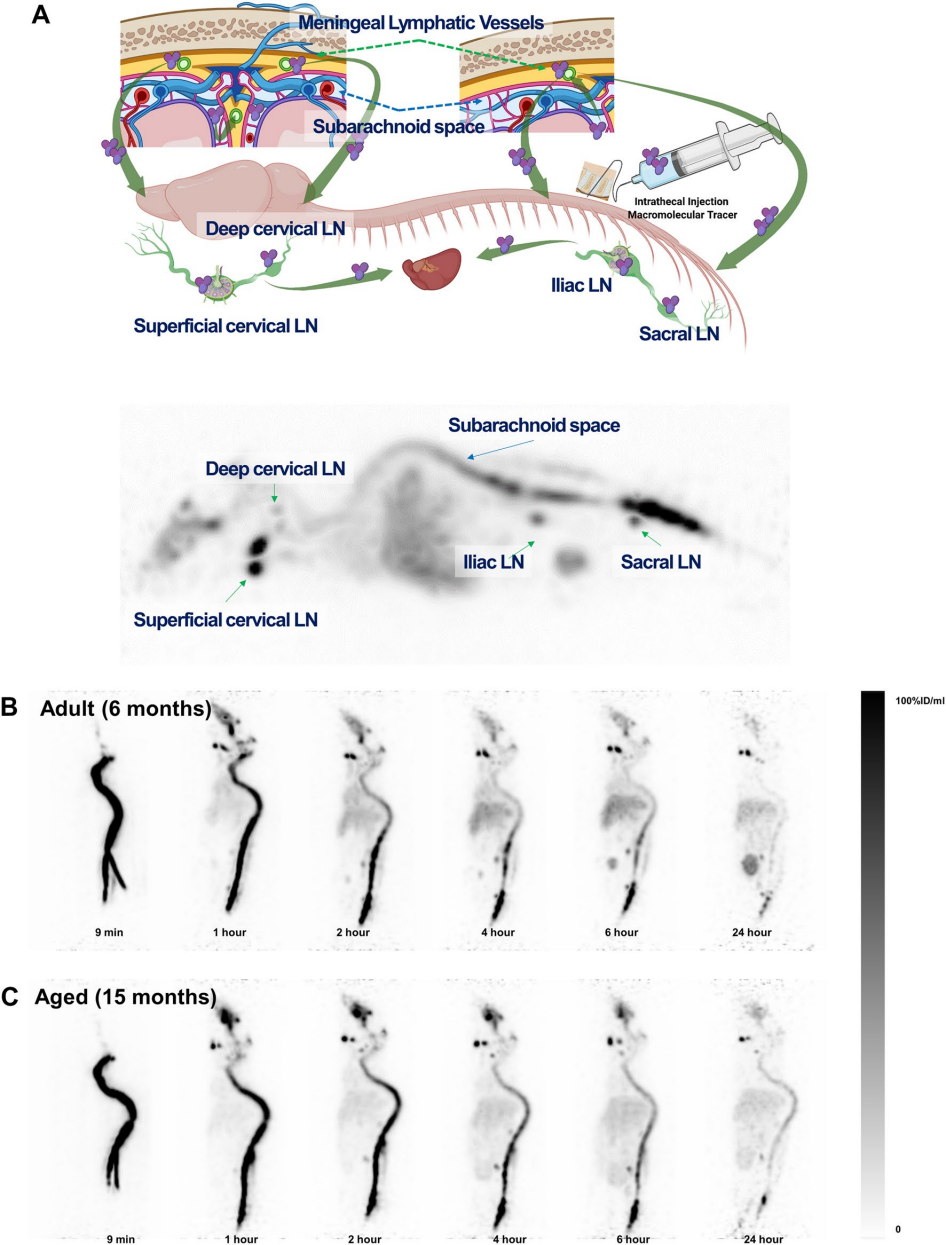
Whole body PET maximum intensity projection images after intrathecal injection of [^64^Cu]Cu-albumin showing the organs involved in the bio-distribution of tracer (**A**), the temporal change of biodistribution in an adult mouse (n = 7) (**B**), and in an aged mouse (n = 6) (**C**). The shaded bar on right of (**B,C**) indicates the activity concentration of the tracer.

On maximal intensity projection (MIP), the animation image of a mouse (Supplementary Movie 1) shows that [^64^Cu]Cu-albumin reached directly the cervical lymph nodes, the pelvic lymph nodes and even farther, via the heart and probable systemic circulation, the liver (Fig. 1A).

MIP animation images of seven adult mice and six aged mice were assessed visually. Individual representative images are shown in Fig. 1B, C and Supplementary Movie 2. In the 9-min images, [^64^Cu]Cu-albumin activity was intense in the spinal subarachnoid space, with negligible tracer concentration in the cranial and most caudal parts of the subarachnoid space, which were later filled in images of both adult and aged mice (Fig. 1B, C).

On the 1-to-6 h images of adult mice (Fig. 1B), radioactivity cleared gradually from the cervical, thoracic and lumbar subarachnoid spaces in adult mice, whereas the hepatic activity continued to increase. At 24 h, the subarachnoid space radioactivity of these adult mice was nearly cleared, with sustained retention in the nasal region, cervical lymph nodes, iliac and sacral lymph nodes, and sacral subarachnoid space. In contrast, on the 1-to-6 h images of aged mice (Fig. 1C), radioactivity cleared more slowly from the cervical, thoracic and lumbar subarachnoid space than in adult mice. Their hepatic activity continued to increase, but intensity was lower in aged mice than in adult mice.

At 24 h, the retained activity within the subarachnoid space of the aged mice was higher than that of adult mice. Radioactivity in the nasal region, cervical lymph nodes, iliac and sacral lymph nodes, and sacral subarachnoid space was similar between adult mice and aged mice. Sacral lymph node activity was faint in aged mice, whereas the subarachnoid space activity was higher in aged mice than in adult mice on visual assessment.

### Quantitative comparison of clearance from the subarachnoid space of intrathecal [^64^Cu]Cu-albumin between adult and aged mice

**Figure 2:**
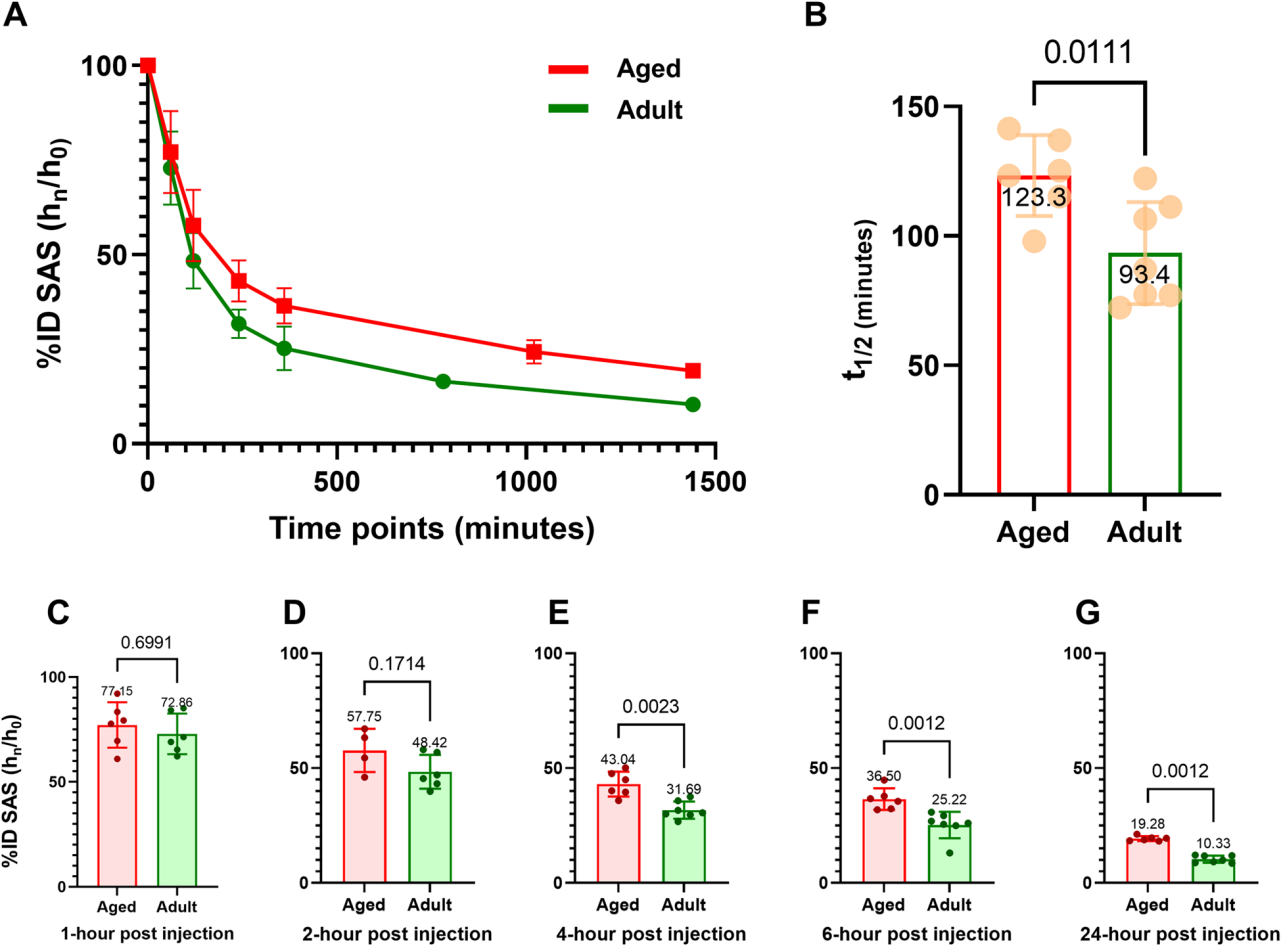
Comparison between adult (n = 7) and aged (n = 6) mice showing time-%ID line-plots for clearance from the entire subarachnoid space (**A**), bar-plots with pairwise comparison for clearance halve-lives (**B**), and bar-plots with pairwise comparison for %IDs at the matched time-points (**C–G**).

The time vs %ID line plot (Fig. 2A) showed an overall temporal decline in the %ID from the entire subarachnoid space, representing the ‘lymphatic drainage of CSF’ in both adult and aged mice. The mean clearance half-lives of the adult and aged groups were 93.4 ± 19.7 (72.3–122.2 range) and 123.3 ± 15.6 (97.9–141.4 range) minutes, respectively. The mean clearance half-life for aged mice was significantly higher (Fig. 2B) than that for adult mice (p = 0.01). The %IDs for aged mice at 4, 6, and 24 h post-injection were also significantly higher (Fig. 2C–G) than those in adult mice (p < 0.05). Clearance was slower in aged mice, and retention was higher in the subarachnoid space in aged mice than in adult mice. The clearance parameters of the sub-arachnoid space in individual mice are shown in Supplementary Table 1.

### In-vivo stability of subarachnoid space-staying [^64^Cu]Cu-albumin after intrathecal injection

**Figure 3:**
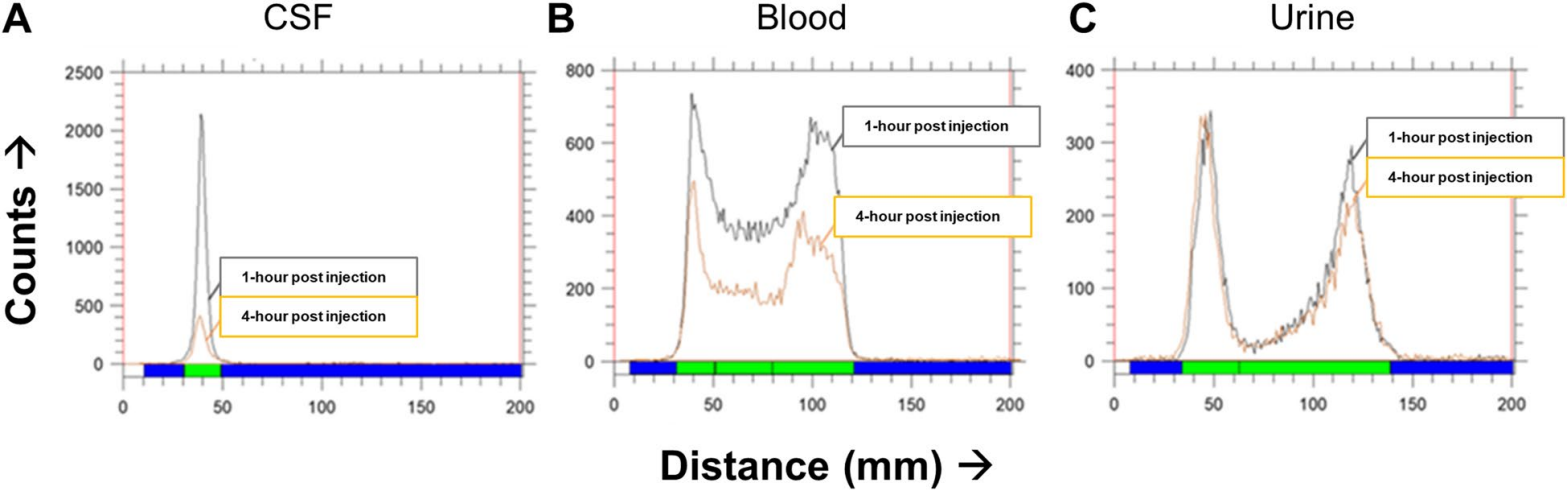
Radioactivity of CSF at 1 h and 4-h following the intrathecal injection with chromatograms for the [^64^Cu]Cu-albumin at the corresponding time points show a single peak for the CSF comprising of greater than 90% of the radioactivity at the origin that represented the intact [^64^Cu]Cu-albumin. In contrast, degraded fragments of [^64^Cu]Cu-albumin moved to the solvent front in variable degree for the blood and urine samples 1 or 4 h after intrathecal [^64^Cu]Cu-albumin injection.

On ITLC, intact [^64^Cu]Cu-albumin remains peaked at the origin, and degraded [^64^Cu]Cu-albumin fragments with variable sizes reached the solvent-front to make the second peak, representing detached and unbound [^64^Cu]Cu-NOTA-N_3_. A single peak was observed at the origin for the CSF sample (Fig. 3A**)**, revealing that [^64^Cu]Cu-albumin was in intact form in the subarachnoid space at 1 and 4 h after intrathecal injection. Intact [^64^Cu]Cu-albumin of blood and urine samples peaked at the origin, and the corresponding degraded fragments moved to the solvent-front at 1 and 4 h, with the amount increasing over time lapse (Fig. 3B, C).

## Discussion

In this study, using [^64^Cu]Cu-albumin PET, we corroborated the lymphatic drainage of CSF assessed in previous studies by fluorescent or MRI contrast imaging^12,15–17^ in mice. In addition, we quantitatively measured the early/acute and late phase of this drainage after intrathecal administration of [^64^Cu]Cu-albumin to assess the long-term stationary phase of this drainage. On PET, the brain and its sulci were barely observed with radioactivity; in contrast, the CSF space around the spinal cord and cranial cisterns maintained the radioactivity even at 24 h after injection. Quantitation of the radioactivity of subarachnoid space revealed a higher retention of [^64^Cu]Cu-albumin in aged mice than in adult mice, and CSF-drained activity was sustained at cervical and sacral lymph nodes. Technically, together with the adjusted least amount of intrathecal injection-volume, the latest time point of imaging enabled us to visualize the late stationary phase of lymphatic drainage, eventually revealing the physiologically minute, albeit definite, decrease in CSF drainage from the brain and spinal cord to the adjacent lymph nodes. We propose that the analysis of lymphatic drainage of CSF by [^64^Cu]Cu-albumin PET and its quantitation a day after intrathecal injection is a good tool for evaluating changes in the lymphatic drainage of CSF in mice. We further expect that assessing the lymphatic drainage of CSF by combining intrathecal [^64^Cu]Cu-albumin PET at a later time point with lymph node imaging will be a good alternative to intrathecal contrast MRI CSF imaging^8–11^ as we can use a smaller injection volume and mass of contrast agent for physiologic/pathophysiologic studies in humans.

Brain waste materials contribute to brain aging both physiologically or pathologically (i.e., hemorrhage, neuroinflammation, trauma or neurodegenerative diseases). Obviously, they are first cleared from the brain parenchyma into CSF within cerebral ventricles and subarachnoid space^21,32,35–37^, and then disposed through the cranial and spinal meningeal lymphatic vessels to the systemic lymph nodes^1,2,6,12,15,16,19,23,27,35,38,39^. This distinctive clearance process, running along the entire length of skull and spines, is known as lymphatic drainage of CSF. Because meningeal lymphatics of brain/spinal cords are associated with peri-cranial/vertebral lymph nodes, cervical^16,19,23^ or sacral^27,38^, the pathways of lymphatic drainage of CSF are presumably separate from brain or spinal cord segments. Wastes would take different clearance paths from the brain and spinal cords in their easiest and shortest path, and these paths also function as a neuroimmune interface between the central immune system (i.e., bone marrow) and the central nervous system (brain and spinal cords) regionally^3,4^. However, it remains to be proven whether the flow from CSF to meningeal lymphatics is segmental, that is, whether vertebral segmental routes are taken separately by lymphatic channels in vertebral segmental divisions. According to two recent reports, two possibilities are there. In one suggestion, brain lymphatics flow from the nasal cavity to the deep cervical lymph nodes (as a secondary stop) via superficial cervical lymph nodes and brain meningeal lymphatics directly flow to deep cervical lymph nodes (as primary stop), whereby vertebral lymphatics exit the vertebral segmental subarachnoid space, with the sacral lymph node as a primary stop and the iliac lymph node as secondary stop^27^. In another suggestion, as every vertebra has its epidural lymphatics to the smaller paravertebral lymph nodes, as was indicated by a mouse whole-body vDISCO study with transparent mounting/imaging^30^. At least a long column of many vertebral segments with epidural lymphatics could have connection with the dura outlets and make primary lymph nodes in their vicinity. This distributive drainage paths might also take the role of gates for adaptive immune surveillance against brain/spinal cord-derived waste and debris^38^. A recent report surprisingly showed that meningeal lymphatics were the route for senescent astrocytes to be deployed to the systemic immune system^39^.

In addition to the decreased and delayed drainage of CSF to the lymphatics in aged mice, we also clearly visualized both cervical and sacral lymph nodes. These findings underscored the physiologic meaning of the inter-communication between brain/spinal cord parenchyma and the regional lymph nodes, but how do these lymph nodes communicate with the central immune system, i.e., bone marrow of skull and vertebrae? Cai et al. discovered the venous connection between bone marrow sinus of the skull and venous sinus of the dura^40^, which could act as a good connection path between these two central systems, neuro and immune, of brain/spinal cord parenchyma and bone marrow of skull/vertebrae. Myeloid/lymphoid cells are known to commute between bone marrows of the skull and vertebrae and brain parenchyma via CSF^3,4^. Lymph nodes are the intermediary station of the information contained in the macromolecules or cells from CSF in every level of the brain and spinal cord segments. Kwon et al.^16^ and Ma et al.^27^ explicitly reported the sacral drainage path in animals, and Jakob et al.^38^ visualized the epidural lymphatic channels of vertebrae, which drain CSF solutes to regional lymph nodes^39^. Thus, the paravertebral epidural lymphatic channels to the nearby lymph nodes are now added for every vertebral segment^32^ in addition to the nasal routes and meningeal lymphatics to the superficial and deep cervical lymph nodes. In our study of [^64^Cu]Cu-albumin using PET, segmental CSF-lymphatic drainage was assessed in a snapshot on serial visual reading of the MIP images (Supplementary Movie 2). Via these pathways, macromolecular solutes and senescent/injured brain/spinal cord cells can meet the innate/adaptive immunity of the immune system. Further visualization studies are warranted for a more detailed understanding of the kinetics of CSF contents of this segmental communication, thereby unveiling the meaning of the closed loop of immune surveillance of brain and spinal cord and regional lymph nodes.

PET using [^64^Cu]Cu-albumin enables us to more easily visualize and quantify lymphatic drainage of CSF, thus providing advantages over other imaging modalities. Other radionuclide imaging studies show a relatively high background because radionuclide-labelled radiopharmaceuticals are administered intravenously; however, in this study, we administered the tracer intrathecally, i.e., within a compartment. Intrathecal administration can show the best contrast and has been used for decades in clinical radioisotope voiding cystography and cisternography. No radioactivity is detected in the body, especially in the vicinity, before intrathecal injection of [^64^Cu]Cu-albumin. The high signal-to-noise ratio of intrathecal radioisotope-labelled albumin PET cisternography makes it possible to qualitatively and quantitatively assess small structures such as deep cervical, sacral, or iliac lymph nodes even in mice. Hot radioactivity was found in superficial cervical lymph nodes, reflecting the lymphatic drainage of CSF through the nasal cavity^6,7,17,41^ and the sustained ratio of activities of superficial cervical lymph nodes and deep cervical lymph nodes 9 min and 1/2/4/6/24 h after injection. Cervical lymphatic tracts connect superficial and deep cervical nodes^42^, and cranial meningeal lymphatic vessels can drain directly to deep cervical lymph nodes^1,2,6,7^. Because albumin (65 kDa) was used as a surrogate of extracellular (CSF) tau (ca. 60 kDa) or amyloid beta (100–150 kDa), the sustained radioactivity observed, even at 24 h, in the image of the lymph nodes suggests that pathologic proteins flown out from the CSF to the CNS-sentinel lymph nodes would be exposed to systemic immune surveillance, i.e., antigen-processing/presenting myeloid cells and adaptive immune cells in the lymph nodes.

Various diseases have been associated with dysfunctional lymphatic drainage of CSF in the literature^41,43–52^, including subarachnoid hemorrhage (SAH)^36^ and intracerebral hemorrhage^44^ in mouse models, which showed drainage of erythrocytes and blood solutes via CSF/meningeal lymphatic drainage paths. In contrast, subdural hematoma may^45^ or may not^44^ be drained via meningeal lymphatics. Brain tumors may also clear their components via meningeal lymphatics to cervical lymph nodes to invoke immunosurveillance^46^, and traumatic brain injury has been associated with meningeal lymphatic dysfunction^47^. Neurodegenerative diseases including Alzheimer’s disease (AD) have been linked to lymphatic drainage dysfunction, and deep cervical lymph node ligation^35^ was known to worsen AD pathology in mouse models^49^, which could be reversed by focused ultrasound^50^ or theta burst electrical stimulation of the brain^45^. Lymphatic ablation using chemical Visudyne^25,31,43,52^ also worsened AD or SAH pathology. Furthermore, meningeal lymphatic dysfunction may be associated with vascular endothelial growth factor-C (VEGF-C) and associated lymphangiogenesis abnormality^31,41,46^. The discrepancy might be partly due to the subtlety of meningeal lymphatic dysfunction which mandates a meticulous examination of the lymphatic drainage of CSF. In small animal models such as mouse, we must use the smallest volume of contrast in intrathecal administration, regardless of the contrast material. In this study, we identified the appropriate material, [^64^Cu]Cu-albumin, and volume and injection rate for intrathecal administration.

For the same purpose of understanding the lymphatic drainage of CSF, pioneering studies^1,2,14^ used ex vivo fluorescent microscopy imaging to visualize/quantify paravascular flux/reflux of intra-cisterna magna injected with fluorescent dyes. The researchers found the size dependence of lymphatic drainage of CSF^14,15^, as well as influx through perivascular spaces from the subarachnoid space^19^, which reportedly differ between young, middle age, and old mice. As expected, young mice had deeper perivascular penetration of intra-cisterna magna-injected materials such as sizable fluorescent dye-labelled dextran (3 kDa) or ovalbumin (45 kDa)^19^. These findings were reproduced in a multicenter study authored by Mestre et al.^53^ but challenged by Smith et al. for their mechanistic interpretation of interstitial fluid space drainage^54^. Notwithstanding these competing ideas of convection/advection through a glymphatic mechanism or diffusion across any interstitial fluid space, the perivascular space between basement membranes of glia limitans and capillary endothelium is crucial under physiological conditions for flowing waste/debris from the brain/spinal cord parenchymal interstitial fluid space to the CSF. Studies with intrathecal gadolinium contrasts, mostly gadobutrol (450 Da), conducted in mice or rats, have even highlighted subtle differences in influx to the perivascular space, showing effects on this glymphatic flow in anesthesia or sleep^22,23,26,55–58^. However, unlike initial fluorescent microscopic studies, the perivascular reflux found in these MRI experiments was not so profound, and only shallow infiltration was observed, especially in rats^55–58^.

The major confounders of these studies were intra-cisterna magna injection (despite confirmed normal intracranial pressure on monitoring) versus intrathecal injection and the larger fractional amount of contrast (relatively in mice) versus the smaller fractional amount of contrast (in rats) and larger quantity of fluorescent contrast in ex vivo/ in vivo fluorescent microscopy versus the relatively smaller quantity of contrast (gadobutrol) in MRI. Among these issues, we hypothesized that the volume was the primary confounder. Because we did not observe any activity in the lymph nodes upon ^99m^Tc -DTPA (diethylene triamine pentaacetate single photon emission computed tomography, albumin (65 kDa), in a trace amount, was chosen to be the optimal candidate for most physiological examinations. In our study, we were not able to observe the brain parenchymal perivascular influx on [^64^Cu]Cu-albumin PET, so we gave up on the glymphatic evaluation, especially on the age-related decline of glymphatic or interstitial lymphatic drainage of CSF, which had been studied previously by reversing the physiologic flux upon administration intra-cisterna magna with a larger volume (10 to 15 μL) at a tolerable, but high rate (1 to 5 μL/min) (Supplementary Table 2). We tried to decrease the volume of radiotracer as much as possible at a 0.3 μL/min rate, but we observed stagnation at the intrathecal space of the injection site, with no movement to the basal cistern, disabling to follow the path of lymphatic drainage of CSF to reach the lymph nodes. As previously described, in mice, the optimal rate was 0.7 μL/min for a total volume of 6 μL (Supplementary Fig. 3)^18^.

The lymphatic flow from primary to secondary lymph nodes remains poorly understood. As stated above, just like the superficial and deep cervical lymph nodes, the sacral and iliac lymph nodes appeared at a similar time after intrathecal administration on our serial PET images. Contrary to the interpretation by Ma et al.^27^ that sacral spine CSF flows out to the sacral lymph nodes as a first stop and then to the iliac lymph nodes which is collecting hub, on our study it seemed that the sacral lymph nodes drained the CSF from sacral epidural lymphatics and the iliac lymph nodes drained the CSF from lumbar epidural lymphatics. We suspect that the iliac lymph nodes work as the collecting site of the vertebral segmental lymphatic drainage from the adjacent spines and also the efferent lymph flows from the sacral lymph nodes, as a local leakage of albumin into the lumbosacral tissues. If recapitulated with the following studies on list-mode PET studies on the earlier time points (immediate to 1 h post-injection), this finding will propose the hierarchical structure of pre/paravertebral lymph nodes covering the entire length of cranium and vertebrae with the possible bidirectional communication between the systemic immune system and central nervous system. Hosang et al. recently reported that infectious inflammation of the lungs affected the states of microglia of the thoracic spinal cord^59^. Likewise, the compartmentalized composition between immune structures (lymph nodes) and spinal cord segments and the brain may be the general platform of the periphery to CNS interaction, especially gut with microbiome and brain/spinal cord^4,60,61^. [^64^Cu]Cu-albumin PET will help to scrutinize, as noninvasively as possible, the kinetics of the lymphatic drainage of CSF until 24 h after intrathecal administration, without further manipulation or with repeated imaging as this method revealed that aging-related decline was there in normal aging mice without much variation between individual mice per lymph nodes activity over %ID of CSF space activity at 24 h imaging (Fig. 2G).

Limitation of our current PET study is that it did not show direct evidence that CSF cross arachnoid barrier cells layer to reach dura lymphatic vessels. Instead, it did show intrathecally laden exogenous albumin reach paravertebral lymph nodes. Ma et al.^25^ could not observe apparent uptake of fluorescent dextran into the dural lymphatic vessels within the skull and reasoned that tight junction of arachnoid barrier cell lay would have prevented the drainage. Perineural sheath/lymphatics beneath cribriform plates and spinal nerve root and epidural lymphatics were shown to be the paths of CSF to systemic lymphatics on the classical post-mortem or in vivo/ex vivo ink/dye/contrast studies in animals^62–65^. However, recent anatomy studies by Kutomi et al.^66^ in pigs and Mollgard et al.^67^ in mice revealed that arachnoid barrier cell layer allows crossing fluid and macromolecules via the gaps such as fissure and cranial arachnoid granulation-like dural gaps in pigs^66^ or subarachnoid villus-like structures in mice^67^. Absence of claudin 11, a tight junction molecule of arachnoid barrier cell layer around juxtaposed subarachnoid lymphatic-like membrane (SLYM) and sagittal sinus vein of cranial dura^67^. At least in mouse, we assume that CSF fluid and/or macromolecules pass through arachnoid barrier cell layer gaps to reach dura lymphatic vessels. As pigs were already shown to have possible pass through arachnoid granulation-like gaps, monkey/primate/human studies remain. Age-related decline of meningeal lymphatic vessels would also be accompanied by the well-known pathology of systemic lymphatic vessels such as (1) decreased lymphatic vessel contractility, (2) basal hyperpermeability and thus reduced inflammatory responsiveness and (3) delayed immune response^68^. Meningeal lymphatic vessels and their mechanistic contribution to age-decline of CSF-lymphatic drainage should be elucidated further.

In the future, cause and effect relationships and their therapeutic significance should be tested by repeatedly imaging small animals under physiological conditions using sophisticated PET/ computed tomography (CT) or PET/MRI. These methods use small quantities of radiopharmaceutical, avoiding any concern about toxicity or perturbation of the stationary state of animals. Animal models of traumatic brain injury, intracerebral/subarachnoid hemorrhage or even aging/sleep/circadian cycles should be accompanied at various levels, degrees of a severity or time points to model disease course in individual animals. Neuroinflammation or dementia models should also be observed repeatedly during their course of disease even at 3/ 7/ 12/ 18 months of age. For these studies at multiple time points, [^64^Cu]Cu-albumin PET is recommended when seeking to understand the physiological and pathological mechanisms of the lymphatic drainage of CSF as the producer of major candidate parameter of a disease course and/or intervention outcomes. The clearance half-life and the surrogate of CSF retention, %IDs at 4, 6 or 24-h post injection are proposed as a marker of CSF drainage abnormality in line with evidence of such an age-related phenotypic change suggesting a dysfunctional clearance of brain waste^25^. Lymph node activities on [^64^Cu]Cu-albumin PET from earlier and late stationary phase is another interesting finding ensuring that [^64^Cu]Cu-albumin PET is showing lymphatic drainage at every level of skull and vertebrae. These biomarkers and the corresponding protocols can be applied to assess the response to novel therapeutic strategies known to enhance the lymphatic drainage of CSF^50–52,69^.

## Conclusions

Aged mice show a significantly higher clearance half-life and tracer retention within the subarachnoid space than adult mice. The imaging parameters of the PET protocol used in this study can be applied to evaluate lymphatic dysfunction and the response after therapeutic enhancement of lymphatic function.

### Supplementary Information

Supplementary Video 1.

Supplementary Video 2.

Supplementary Information.

## Data Availability

All data are available in the main text or supplementary materials. All data, code, and materials used in the analysis are available through a standard material transfer agreement with Seoul National University for academic and nonprofit purposes by contacting the corresponding authors (dsl@snu.ac.kr or mandu3710@gmail.com).
