# Supplementary material for: Intrathecal [64Cu]Cu-albumin PET reveals age-related decline of lymphatic drainage of cerebrospinal fluid
Source: Sci Rep. 2023 Aug 9;13:12930. doi: 10.1038/s41598-023-39903-y (PMC10412645; doi:10.1038/s41598-023-39903-y)

Supplementary Data

Intrathecal [^{64}Cu]Cu-albumin PET reveals age-related decline of lymphatic drainage of cerebrospinal fluid

List of Supplementary Materials

Supplementary Notes

Our study was approved by IACUC of Seoul National University in 2020 whose approval process was based upon our institutional codes of regulation conduct referring to the recommendations including previous ARRIVE guideline available at that time, however, with the new ARRIVE 2.0 guideline published in 2021, we reviewed our procedure in methods and added the comments according to the ARRIVE Essential 10 (https://arriveguidelines.org)..

Study design

Adult and aged mice were grouped from the same strain of C57BL/6, whose age range were wide enough to cover the range of adulthood but separate from the other at least 6 months difference. This could have reduced variability-related type II error (false negative results).

Sample size

For ethical reasons, we tried to reduce the number of mice as small as possible, but for acquisition of the sufficient number to discover the true difference between groups, we needed to increase the number of mice as large as affordable (in terms of the survival of the mice after intrathecal injection and repeated anesthesia/sedation and imaging in warm blanket within the gantry of PET scanner). Sample size was determined by the prior experiment which established the imaging protocol and injection/anesthesia/mice wellbeing and survival techniques (18).

Inclusion and exclusion criteria

Considering that the repeated imaging procedures after the initial anesthesia/operation/intrathecal injection preclude any possibility of exclusion, the only reason of exclusion was technical failure of experiment despite meticulous handing of entire experimental/imaging procedures. 15-25 months old mice of aged group were the survivors till that age, and thus the survivors in our institutional animal facility taken care of by ourselves and thus survivors. The mice which failed to thrive would have been excluded.

Randomization

Adult mice were randomly selected among the mice we obtained from vendor, and those who survived the aging were also included to the aged group.

Blinding

Blinding between adult and aged mice group could not be blinded to the investigators. Blinding of reading mice PET and of quantification based on drawing of regions of interest (ROIs) on CSF or organs were not done simply because it was not relevant. Retrospectively, the same ROI was transferred from the first image labeled 'immediate' to the images of later time (2h, 4h, … 24h). However, the ROIs should have been adjusted due the position change of mice at each successive imaging epoch.

Outcome measures

Two measures are relevant to say as for outcome measures. First, alternation scores for Y-maze were measured as established standard. Especially acclimation the day before and the measurement ensured the reproducibility and thus the variability of alternation scores per individual mice represent the biological variability of individual mice. Second, individual variability of CSF ROI activity were quite large to obliterate the difference between groups at earlier times (immediate, 2h, 4h) but came to be mitigated at later time (especially 24h).

Statistical measures

Statistical methods using non-parametric comparison of adult and aged groups of mice of seven/six could yield sufficiently low type I error (false positive). As we have measured individual mice six-times repeatedly, entire number of measurement was 78, meaning 78 volume-type whole body PET images available for several ROI quantification. As this study was exploratory one that the hypothesis was that there would be difference between adult and aged mice and as we found the difference in CSF clearance and lymph node uptakes along time after intrathecal injection, we now can make posterior hypothesis that there might be difference between aged normal controls and young disease (for example, AD model such as 5xFAD) model mice with of without novel treatment. In this further experiment, we might use lymph node to CSF ratio at 24h PET as a parameter to reveal the effect of novel disease-modifying treatment.

Experimental animals

We used a single strain normal mice whose strain was C57BL/6 and only male mice. This was because our contingent experiment was about the reduced lymphatic drainage of CSF in male 5xFAD mice. 5xFAD mice are derivative of C57BL/6 strain. Female mice and their aging-related effect on CSF-lymphatic drainage is open to further investigation.

Experimental procedures.

Technical establishment was reported in our previous publication and in the main text, especially for the handling of radiopharmaceutical and radioligand for labeling and stability in mice in vivo after intrathecal injection.

Results

Details of results are referred to the main text. To avoid redundancy and to maintain succinctness of description we would not comment any further here in this supplementary note.

Supplementary Table 1. Clearance parameters of individual mice after intrathecal [^{64}Cu]Cu-albumin administration from subarachnoid space

Supplementary Table 2. Infusion speed and volumes for tracer infusion in the CSF of mice reported in the literature

Supplementary Figure 1. Delineation of Subarachnoid Space (SAS).

Supplementary Figure 2. Alternation scores (%) of adult (n=9) (2-9 months old) and aged mice (n=9) (15-25 months old) for spatial memory on a Y-maze test

Supplementary Figure 3. Ex-vivo distribution of Evans blue dye after intrathecal injection at different infusion speeds and volumes a) 0.3 µL/min, 3 µL, b) 0.5 µL/min, 3 µL, c) 0.6 µL/min, 3 µL, d) 0.7 µL/min, 3 µL, and e) 0.7 µL/min, 6 µL. Evans blue dye in 2% in artificial CSF was injected in adult mice; photographs were taken after cardio-perfusion with phosphate-buffered saline.

Supplementary Movie 1. Whole body PET maximum intensity projection images after intrathecal injection of [^{64}Cu]Cu-albumin showing the organs involved in the bio-distribution of tracer.

Supplementary Movie 2. Whole body PET maximum intensity projection images after intrathecal injection of [^{64}Cu]Cu-albumin showing the temporal change of biodistribution in an adult mouse (A), and an aged mouse (B).

Supplementary Table 1. Clearance parameters of individual mice after intrathecal [^{64}Cu]Cu-albumin administration from subarachnoid space

	Half-Life (min)	Plateau (%ID)	K (min^{-1})
Adult mice			
(n=7)	106.6	15.28	0.006501
	87.14	13.43	0.007954
	72.29	16.16	0.009588
	77.36	14.78	0.008959
	122.2	14.46	0.00567
	111.2	15.77	0.006232
	77.05	13.78	0.008996
Aged mice			
(n=6)	115.2	24.47	0.006017
	141.4	19.55	0.004903
	123.3	21.22	0.005619
	97.97	20.61	0.007075
	125.2	23.65	0.005536
	136.9	19.63	0.005062

Supplementary Table 2. Infusion speed and volumes for tracer infusion in the CSF of mice reported in the literature

Publication	Method	Route	Total volume (µL)	Rate (µL/min)
Wang 2020 Sci Transl Med (70)	EVFM	ICM	15	1.5
Aspelund 2015 J Exp Med (2)<br>Hablitz 2019 Sci Adv (55)	EVFM	ICM	10	2
Iliff 2012 Sci Transl Med (12)<br>Iliff 2013 J Clin Inv (71)<br>Kress 2014 Ann Neurol (19)<br>Smith 2017 eLife (54)	IVTPM	ICM	10	2
Iliff 2014 J Neurosci (72)	EVFM	ICM	10	1
Xue 2020 Sci Rep (23)	MRI	ICM	7	1
Xie 2013 Science (73)<br>Achariyar 2016 Mol Neurodegener (74)	IVTPM	ICM	5	1
Louveau 2015 Nature (1)	EVFM			
Ma 2017 Nat Commun (15)	NIRI			
Da Mesquita 2018 Nature (5)	MRI	ICM	2-5	2.5
Jacob 2019 Nat Commun (38)	EVFM	ICM	2	0.5
Gaberel 2014 Stroke (75)	MRI	ICM	1	1
Wu 2018 J Nanobiotechnology (76)	IVFI	IT	10	Bolus
Jacob 2019 Nat Commun (38)	EVFM	IT	2 and 8	1
**This study**	**RI**	**IT**	**6**	**0.7**

EVFM *Ex vivo* Fluorescent Microscopy, IVTP *In vivo* Two Photon Microscopy, MRI Magnetic Resonance Imaging, NIRI Near Infrared Imaging, IVFI *In vivo* Fluorescence Imaging, ICM Intra Cisterna Magna, IT Intrathecal, RI Radionuclide Imaging

Supplementary Figure 1. Delineation of Subarachnoid Space (SAS). Delineation of SAS was conducted based on the analysis of a one-hour image. This process was carried out separately at the spinal cord level and the skull level. At the spinal cord level, the center of the SAS was determined through visual inspection of the transaxial images, and a circular Region of Interest (ROI) with a diameter of 6 mm was established. The SAS was then segmented in each transaxial slice using a subthreshold of 10%, chosen to account for the spillover effect, of the maximum Standardized Uptake Value (SUVmax). These segmented ROIs were interpolated to create a comprehensive 3-dimensional representation of the spinal SAS. At the skull level, the center of the skull cross-section was identified through visual inspection of the transaxial images, and a circular ROI with a diameter of 10 mm was placed. Following the same subthresholding and interpolating steps as described above, we constructed a comprehensive 3-dimensional representation of the cranial SAS. These Volume of Interest (VOI) results were then uniformly applied across various time frames. Where registration mismatches occurred, manual corrections were implemented to ensure accuracy.

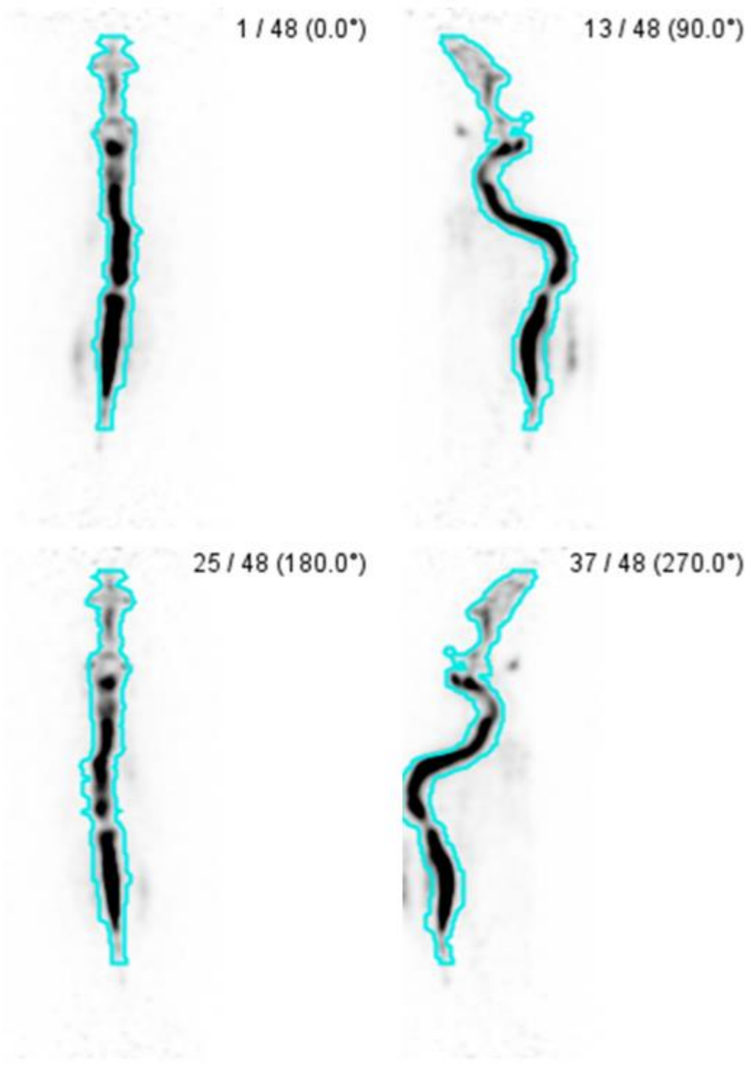

Supplementary Figure 2. Alternation scores (%) of adult (n=9) (2-9 months old) and aged mice (n=9) (15-25 months old) for spatial memory on a Y-maze test

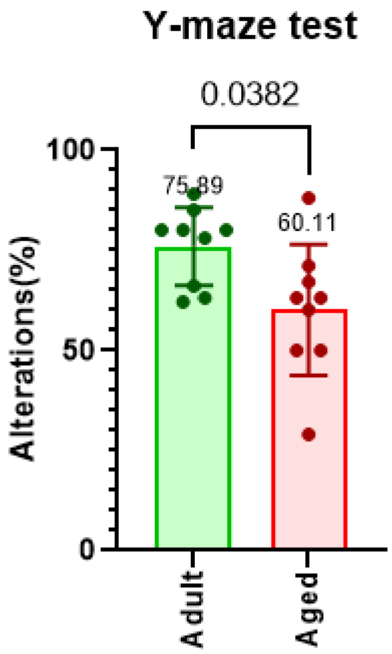

Supplementary Figure 3. Ex-vivo distribution of Evans blue dye after intrathecal injection at different infusion speeds and volumes a) 0.3 μL/min, 3 μL, b) 0.5 μL/min, 3 μL, c) 0.6 μL/min, 3 μL, d) 0.7 μL/min, 3 μL, and e) 0.7 μL/min, 6 μL. Evans blue dye in 2% in artificial CSF was injected in adult mice; photographs were taken after cardio-perfusion with phosphate-buffered saline.

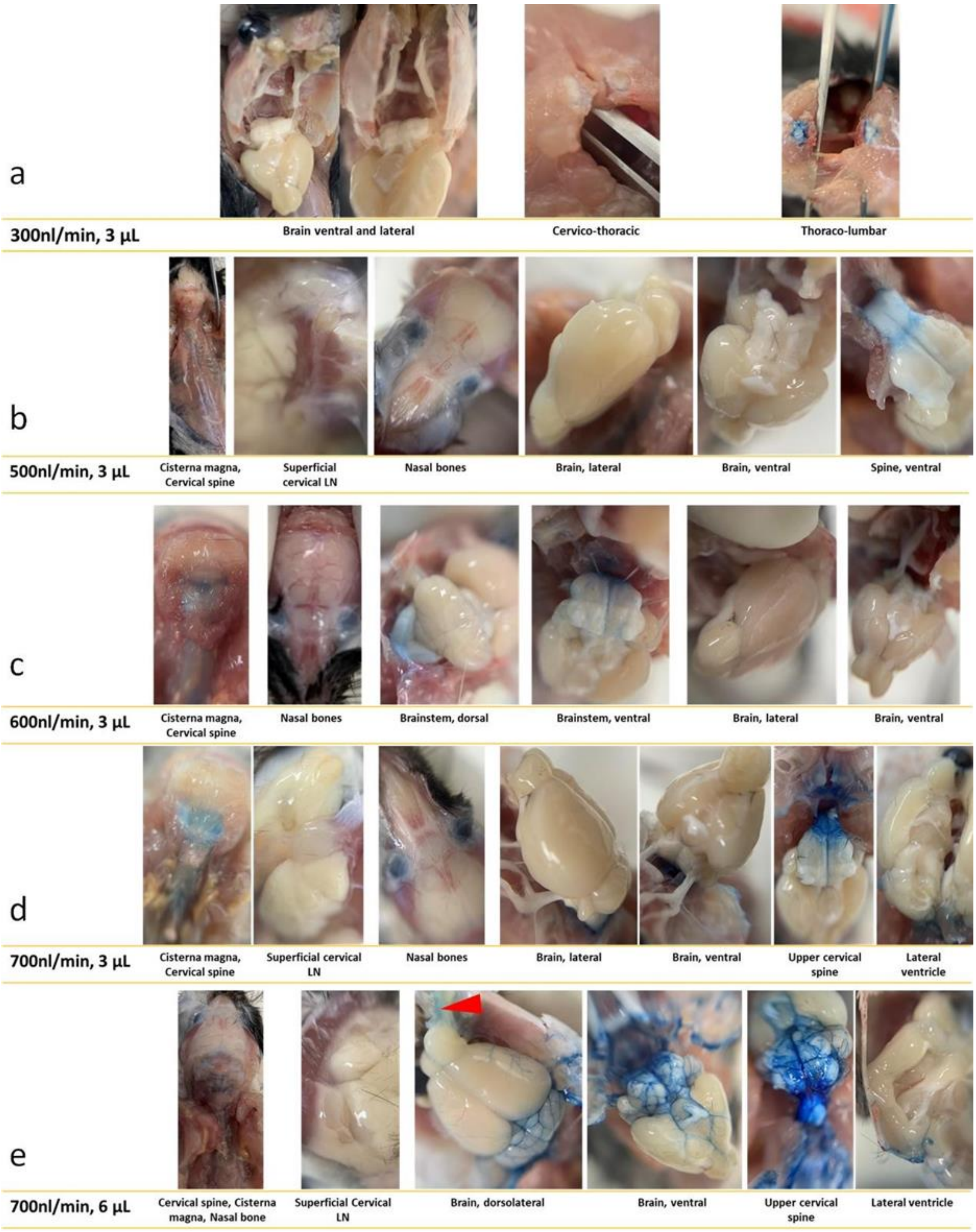

Supplement: Supplementary file 3 — Supplementary Information. [file 41598_2023_39903_MOESM3_ESM.pdf]
